# Molecular Characterization of Carbapenem Resistant *Klebsiella pneumoniae* in Malaysia Hospital

**DOI:** 10.3390/pathogens10030279

**Published:** 2021-03-02

**Authors:** Min Yi Lau, Fui Enn Teng, Kek Heng Chua, Sasheela Ponnampalavanar, Chun Wie Chong, Kartini Abdul Jabar, Cindy Shuan Ju Teh

**Affiliations:** 1 Department of Medical Microbiology, Faculty of Medicine, University of Malaya, Kuala Lumpur 50603, Malaysia; minyi@um.edu.my (M.Y.L.); tfenn@ummc.edu.my (F.E.T.); 2 Department of Biomedical Science, Faculty of Medicine, University of Malaya, Kuala Lumpur 50603, Malaysia; khchua@um.edu.my; 3 Department of Infectious Diseases, University Malaya Medical Centre, Kuala Lumpur 50603, Malaysia; sheela@ummc.edu.my; 4 School of Pharmacy, Monash University Malaysia, Subang Jaya 47500, Malaysia; chong.chunwie@monash.edu; 5 Centre for Translational Research, Institute of Research, Development and Innovation, Kuala Lumpur 57000, Malaysia

**Keywords:** carbapenem-resistant *Klebsiella pneumoniae*, *Enterobacteriaceae*, OXA-48, NDM, carbapenemase, PFGE

## Abstract

The emergence of carbapenem-resistant *Klebsiella pneumoniae* (CRKP) is a great concern, as carbapenems are the last-line therapy for multidrug-resistant Gram-negative bacteria infections. This study aims to report the epidemiology of CRKP in a teaching hospital in Malaysia based on the molecular genotypic and clinical characteristics of the isolates. Sixty-three CRKP strains were isolated from a tertiary teaching hospital from January 2016 until August 2017. Carbapenemase genes were detected in 55 isolates, with *bla*_OXA-48_ (63.5%) as the predominant carbapenemase gene, followed by *bla*_NDM_ (36.5%). At least one porin loss was detected in nine isolates. Overall, 63 isolates were divided into 30 clusters at similarity of 80% with PFGE analysis. Statistical analysis showed that in-hospital mortality was significantly associated with the usage of central venous catheter, infection or colonization by CRKP, particularly NDM-producers. In comparison, survival analysis using Cox proportional hazards regression identified a higher hazard ratio for patients with a stoma and patients treated with imipenem but a lower hazard ratio for patients with NDM-producing CRKP. OXA-48 carbapenemase gene was the predominant carbapenemase gene in this study. As CRKP infection could lead to a high rate of in-hospital mortality, early detection of the isolates was important to reduce their dissemination.

## 1. Introduction

Carbapenems are often used as the last line defence to treat nosocomial infection caused by extended spectrum β-lactamases (ESBL) producing Gram-negative bacteria. However, selective pressure from the overuse or misuse of carbapenems has resulted in the emergence of carbapenem-resistant *Enterobacteriaceae* (CRE). Within the increasing number of reported CRE cases worldwide, carbapenem-resistant *Klebsiella pneumoniae* (CRKP) takes precedence as the predominant isolate [1,2]. The first CRKP was detected in North Carolina, USA, in 1996, and the pathogen further spread to new geographical areas over the years [3,4]. The rapid and global emergence of CRKP is concerning, as CRKP is resistant to nearly all antibiotics, including carbapenems, putting severe limitations on the choice of antimicrobials.

Carbapenem resistance in CRKP is mainly by means of the production of specific carbapenem-hydrolysing β-lactamases known as carbapenemases, and the production of either cephalosporinases (AmpC β-lactamases) or ESBLs with a very low level of carbapenem-hydrolysing activity combined with decreased permeability due to porin loss or alteration [5]. Carbapenem resistance in *K. pneumoniae* is mainly the result of the acquisition of carbapenemase genes through the horizontal transfer of resistance plasmids, conjugation being the most common means of transfer [6]. Carbapenemase-producing *K. pneumoniae* harbour genes expressing carbapenem-hydrolysing β-lactamases that inactivate carbapenems, conferring resistance not only to carbapenems but also to most β-lactams, such as penicillins and cephalosporins, including broad-spectrum cephalosporins. Some of the most clinically important carbapenemases are *Klebsiella pneumoniae* carbapenemases (KPC), imipenemases (IMP), Verona integron-encoded metallo-β-lactamases (VIMs), New Delhi metallo-β-lactamases (NDMs) and oxacillinase-48 (OXA-48) [7].

In Malaysia, resistance to carbapenems, such as imipenem and meropenem, in *K. pneumoniae* was documented as early as 2003 by National Surveillance of Antimicrobial Resistance (NSAR), which conducts the surveillance of antimicrobial resistance in the country by collecting data on antibiotic resistance from microbiology laboratories from all 13 states in Malaysia. The first reported CRKP in Malaysia was in June 2004, where a CRKP that was resistant mainly to imipenem was detected [8]. The carbapenem resistance observed was the result of reduced permeability due to the loss of *ompK*36 outer membrane protein with AmpC hyperproduction. Within the next few years, more reports on CRKP in different states in Malaysia began to surface, the majority of which were carbapenemase-producing *K. pneumoniae* that harboured carbapenemase genes, such as NDM-1 [9,10,11,12,13], IMP-4 [10,13], OXA-232 [12], OXA-48 [14] and KPC-2 [14]. In spite of this, published data on the epidemiology of CRKP in Malaysia remained scarce. To address the significant data gaps in the epidemiology of CRKP in Malaysia, we evaluated the incidence of CRKP in a teaching hospital between January 2016 and August 2017. The potential risk factors for in-hospital mortality were further identified using a combination of logistic regression and survival analysis.

## 2. Results

### 2.1. Overview of Carbapenem Resistant Klebsiella pneumoniae Cases

Between January 2016 and August 2017, 63 patients were infected with CRKP in UMMC. An average of three new CRKP cases were detected every month. A total of 40 male and 23 female patients of Malay, Chinese and Indian descent and other races were included. The mean age was 49.55 years, with the youngest patient being a 1-month-old baby, and the eldest being an 85-year-old patient. 

Clinical data for 60 out of 63 patients were successfully extracted and included in the analysis (Table 1). Among the 60 patients, 26 (43.3%) died during hospitalization, whereas the remaining 34 subsequently recovered and were discharged from the hospital. The average length of hospitalization was 53.2 days. More than half of the patients (58.3%) were admitted to the ICU. All of the patients had contact with invasive devices, such as an indwelling urinary catheter, mechanical ventilator, tracheostomy, central venous catheter, art line, peripheral venous catheter, nasogastric tube and stoma (Table 1). Almost half of the patients had common comorbidities, such as chronic kidney disease (13.3%), diabetes mellitus (31.7%) and hypertension (33.3%). A total of 81.7% of the patients had polymicrobial infection involving other bacteria, such as *Escherichia coli*, *Acinetobacter baumanii* and *Staphylococcus aureus* in addition to CRKP. In nearly all of the patients, in-hospital mortality was due to sepsis and pneumonia.

Approximately 95% of patients had been prescribed antibiotics for 90 days prior to CRKP isolation. Thirty-six patients (60%) were given empiric treatment when bacteremia symptoms were detected. All CRKP strains investigated were resistant to amoxicillin-clavulanate; ampicillin-sulbactam; ampicillin; ertapenem; second-generation cephalosporins and third-generation cephalosporins, including cefuroxime, ceftazidime, ceftriaxone and cefotaxime. The proportions of strains susceptible to the remaining antibiotics were as follows: meropenem (71.0%), ciprofloxacin (61.9%), trimethoprim-sulfamethoxazole (59.7%), imipenem (57.1%) and gentamicin (52.4%). Two colistin-resistant *K. pneumoniae* were detected. Phenotypic carbapenemase detection by MHT was performed on the CRKP strains. Forty-five out of 63 strains were MHT positive. Among the remaining MHT-negative strains, 12 harboured carbapenemase genes.

Among the 60 patients, 36 (60%) had active CRKP infections, whereas 23 (38.3%) were colonized with CRKP, as indicated by the absence of clinical and biochemical evidence of an infection; thus, these patients were not given any antimicrobial therapy. In total, 50% (*n* = 30) of the CRKP strains were hospital-acquired, while a smaller portion comprised community-acquired infections (6.7%, *n* = 4) and healthcare-associated infections (3.3%, *n* = 2). Furthermore, 26 out of 36 active infection patients received appropriated definitive treatment, which included monotherapy or combination therapy. The most optimal treatment for CRKP infections remains unknown. Here, we defined appropriated definitive treatment as an antimicrobial drug given according to the antimicrobial susceptibility testing report from diagnostic labs for at least 48 h [15,16]. The drugs used for the CRKP treatment of these 63 patients included imipenem, meropenem, aminoglycosides, ciprofloxacin, tigecycline and colistin.

### 2.2. Determination of Carbapenemases and Porin-Associated Genes

The detection of common carbapenemase genes and porin-associated genes was carried out for all CRKP strains as described earlier. Among the 63 carbapenem-resistant strains, 88.8% of the strains harboured carbapenemase genes, with *bla*_OXA-48_ (63.5%) as the predominant carbapenemase gene, followed by *bla*_NDM_ (36.5%). Seven strains harboured multiple carbapenemase genes, which were *bla*_OXA-48_ and _blaNDM_. However, the carbapenemase genes, *bla*_KPC_, *bla*_IMP_ and *bla*_VIM_, were not detected in this study. The loss of porin *ompK*36 or loss of porin *ompK*37 was detected in 9 CRKP strains.

### 2.3. Genetic Relatedness between CRKP Strains

**Figure 1:**
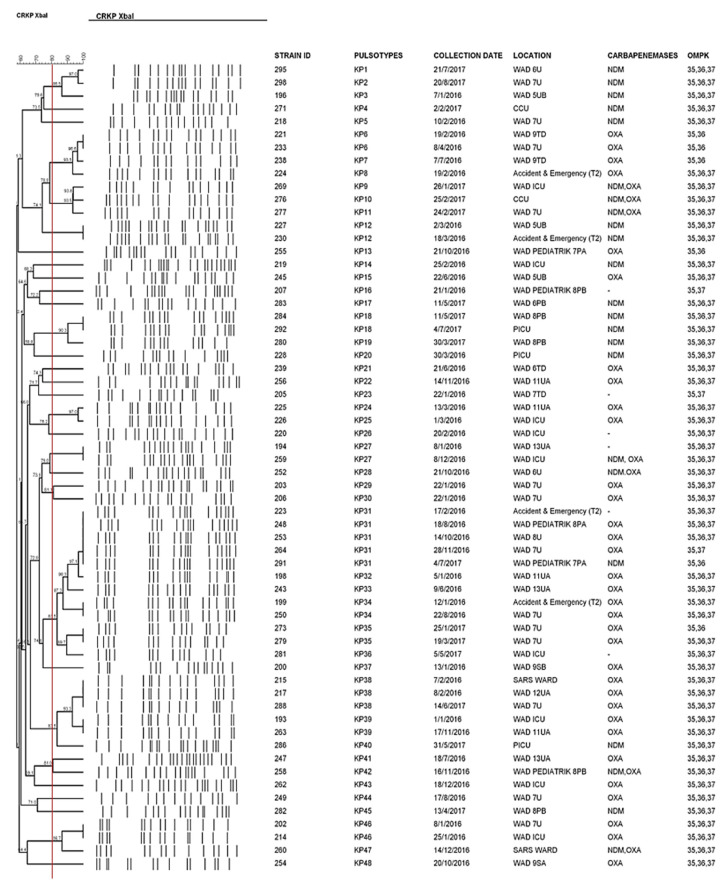
Dendrogram generated from Bionumeric software version 7.6.2 showing the PFGE profile of carbapenem-resistant *Klebsiella pneumoniae* together with their pulsotypes, collection date, ward and genomic profiles. Red line refers to the 80% similarity.

Molecular typing by PFGE analysis was performed on the 63 CRKP strains. Excluding one strain which was not typeable, all remaining CRKP strains were classified into 48 pulsotypes with KP31 as the predominant pulsotype (Figure 1). However, strains grouped under this pulsotype did not cause any outbreak in our hospital, as they were collected from a different timeline. Based on 80% PFGE pattern similarity, 62 CRKP strains were grouped into 30 clusters, suggesting the high diversity of the CRKP clones that emerged in our local setting.

### 2.4. Statistical Analyses

Based on the firth logistic regression, the usage of invasive devices, such as a central venous catheter (OR 12.90, 95% CI 2.23–210.37); colonization (OR 0.02, 95% CI 0.00053–0.17); or hospital acquired infection (OR 0.26, 95% CI 0.17–1.66) by CRKP and the presence of NDM carbapenemase gene (OR 0.081, 95% CI 0.0067–0.43) were significant confounding factors for in-hospital mortality. 

**Figure 2:**
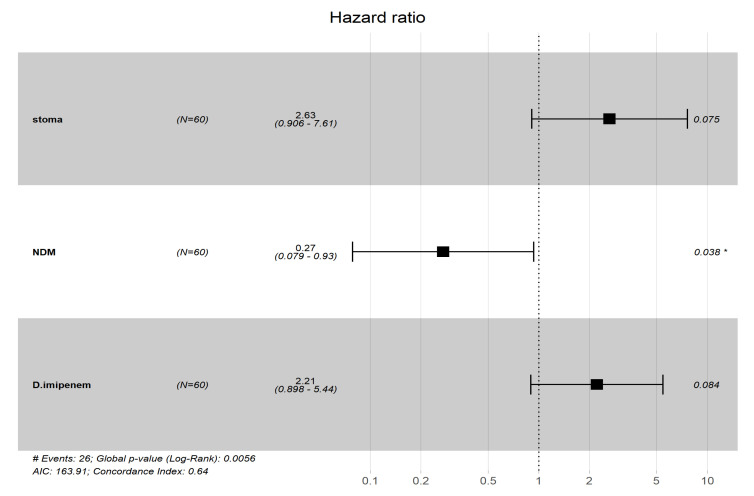
Forest plot generated from R. Hazard ratio less than 1 indicated longer survival time. * denoted *p* < 0.05.

A complementary Cox proportional hazards model was conducted to evaluate the association of risk factors with survival time. The presence of OXA carbapenemase gene, NDM carbapenemase gene, colonization with CRKP, patients with a stoma, previous exposure to third-generation cephalosporins and definitive treatment with imipenem were found to be significant with *p* < 0.15 among all univariate models (Table 2). In the multivariate Cox analysis, the combination of a stoma, NDM producer and imipenem was selected. Specifically, patients with a stoma who received imipenem as definitive treatment showed a higher risk of mortality, while patients harbouring CRKP with NDM showed longer survival (Figure 2).

## 3. Discussion

CRKP has disseminated in many countries across the world over the past decade, Malaysia not being an exception. The incidence of CRKP detection in our hospital setting continuously increased from 17 reported CRKP cases in 2013 to 46 reported CRKP cases in 2016 [14]. An average of three new CRKP cases were detected each month during this study period. This was relatively high when compared to the reported cases in a previous study conducted by Low et al. [14].

Our findings revealed that the majority of CRKP isolates from UMMC showed carbapenemase-mediated resistance with 88.8% of CRKP strains shown to harbour carbapenemase genes. Prior to the molecular detection of carbapenemase genes by PCR amplification, MHT for the phenotypic detection of carbapenemases was carried out. MHT was negative for 18 out of the 63 strains tested. However, upon screening of carbapenemase genes by PCR amplification, 12 strains were found to harbour carbapenemase genes. It is noted that MHT produces false-negatives in carbapenemase-producing isolates of *Enterobacteriaceae*, and at the same time, false-positives are frequently seen in isolates that produce high levels of AmpC β-lactamases (cephalosporinases) or CTX-M-type ESBLs [4].Our findings provide further evidence on the low sensitivity and specificity of MHT, especially for NDM producers, as described in previous studies [15,16,17]. Although MHT has been widely used in the clinical laboratory for carbapenemase detection, it is unable to identify the class of carbapenemase genes involved. Identification of the class of carbapenemases gene in CRKP is important for therapeutic decision making. AST results alone sometimes may be sufficient for the selection of antimicrobial therapy, but different antibiotics may exhibit varying levels of susceptibility to the action of carbapenemases [18]. Some carbapenemases are capable of hydrolysing carbapenems very efficiently, while others may do so to a lesser extent. Some carbapenemases are also active against broad-spectrum cephalosporins, while others are not. [19]. For example, class B metallo β-lactamases (NDM, IMP and VIM) inactivate most of the available β-lactams [1]. Class A carbapenemase (KPC) is able to hydrolyse nearly all β-lactams, including carbapenem, penicillins, aztreonams and cephalosporins, but it is inhibited by β-lactamase inhibitors, especially clavulanic acid and tazobactam [20]. OXA-48 variants have different susceptibility towards carbapenem. For example, OXA-181 hydrolyses carbapenems more effectively as compared to OXA-48, whereas OXA-232 shows lower hydrolytic ability toward carbapenems as compared to OXA-48 [21]. However, the presence of carbapenemase genes sometime does not confer resistance towards carbapenems. For example, most OXA-type carbapenemases exhibit reduced susceptibility to carbapenems but become resistance when there is a combination of carbapenemase activity and secondary mechanisms, such as reduced outer membrane permeability, increased efflux pumps or hyperexpression of broad-spectrum cephalosporinase [22]. These secondary mechanisms have also been associated with the resistance of a non-carbapenemase-producing strain [23]. Hence, the treatment decision should be made based on both genotypic and phenotypic characteristics to ensure a better patient outcome.

Molecular detection by PCR is the standard for the identification of carbapenemases with the use of established primers. It also provides much needed advantages of specificity and sensitivity, which MHT cannot offer. The 63 CRKP strains included in this study were screened for the presence of carbapenemase genes via PCR amplification. The majority of the strains harboured OXA-48 gene (63.5%); this finding is in concordance with the report by Low et al. on carbapenemase gene detection in our hospital setting in 2013 [14]. This is not surprising, as genetic analyses have indicated that the gene that encodes OXA-48 has the propensity to spread among the enterobacterial species at a much higher rate as compared to KPC and NDM genes [4]. Of note, the prevalence of OXA-48 gene may well be underestimated, as isolates that harbour *bla*_OXA-48_ can retain their susceptibility to carbapenems, thus remaining undetected, creating opportunities for the silent dissemination of this gene via a single 62-kb IncL/M-type plasmid pOXA-48a to other bacterial populations [24,25]. This may possibly account for the prevalence of *bla*_OXA-48_-positive CRKP in our hospital setting. 

NDM gene was detected in the remaining 36.5% of CRKP strains. This showed that there was an increase in the prevalence of NDM genes among CRKP strains in UMMC, as a study on carbapenemase gene detection in our hospital setting in 2013, which was conducted by Low et al., reported only one *bla*_NDM_-positive CRKP among all the tested strains [14]. The increased prevalence of *bla*_NDM_ among the CRKP strains implied that the high transmissibility and plasticity of *bla*_NDM_ positive plasmids [26] might have contributed substantially to its spread among existing bacterial populations in UMMC. This was evidenced by reports of highly heterogenous *bla*_NDM_-containing plasmids that varied in molecular size and incompatibility type being found in a wide range of species and genera of Gram-negative bacteria, which, even within individual species, have been shown to be diverse [27]. This inter-strain, inter-species and inter-genus transmission of the *bla*_NDM_-containing plasmids indeed plays a significant role in the epidemiological spread of *bla*_NDM_ in Gram-negative bacteria [27]. 

Among the metallo-β-lactamases found in *Enterobacteriaceae*, NDM has been reported to be the most widely spread [28]. Importation of *bla*_NDM_-positive bacteria from India may contribute to the increased prevalence of *bla*_NDM_-positive CRKP due to the historical link between Malaysia and India. The Indian subcontinent serves as an important reservoir of *bla*_NDM_-positive bacteria, which have been detected in 10 areas in India, eight areas in Pakistan and one area in Bangladesh [19,27]. Within multiethnic Malaysia, tourism aside, travel to India is an ever-present and inevitable factor to consider due to the number of Malaysians of Indian descent and even migrant workers who make trips back to the home country of their ancestors. This precedes the importation of *bla*_NDM_-positive bacteria via asymptomatic colonization from the NDM-endemic Indian subcontinent, where even public drinking water has been found to be contaminated with NDM-producing CRKP [29]. This was exemplified in a published report by Al-Marzooq et al., in which a CRKP harbouring *bla*_NDM-1_ and *bla*_OXA-232_ was isolated from a Malaysian who had returned from India [30]. 

CRKP isolates may harbour more than one carbapenemase gene. In this study, seven CRKP strains co-harboured *bla*_OXA-48_ and *bla*_NDM_, which was not surprising because plasmids that carry *bla*_NDM_ have been associated with other carbapenemase genes, such as *bla*_OXA-48_ and *bla*_VIM_ [4]. It has been documented that such isolates may show a high level of carbapenem resistance, as reported by J. Yan et al. in a study conducted at a tertiary teaching hospital in Chongqing, China, where CRKP isolates that harboured dual carbapenemase genes (*bla*_NDM-1_ and *bla*_KPC-2_) had dramatically higher MIC ranges for ertapenem, meropenem and imipenem [31]. Such isolates may cause infections that are associated with high mortality rates in patients due to treatment complications. 

Of the 63 CRKP strains tested, *bla*_KPC_, *bla*_IMP_ and *bla*_VIM_ were not found in any of the isolates. Based on limited data from previous reports on Malaysian CRKP isolates, *bla*_KPC-2_ has been detected (8 out of 17 CRKP isolates) from a tertiary teaching hospital [14], while *bla*_IMP-4_ has been detected (6 out of 13 CRKP isolates) from Hospital USM in Kelantan [10]. There was no report on the detection of *bla*_VIM_ in Malaysian CRKP isolates, although these metallo-β-lactamase (MBL) genes (*bla*_VIM-2_ and *bla*_IMP-1_) have been recently described in P. aeruginosa isolates in Malaysia [32]. The prevalence of *bla*_IMP_ might not be effectively reflected in the present study, as this gene can also be found in carbapenem-sensitive strains, which were excluded in this study. The weak hydrolytic effect of IMP hydrolase on carbapenems accounts for carbapenem-sensitive phenotypes [33]. This was evidenced in a study conducted in Taiwan where 35 out of 40 *bla*_IMP_-positive isolates of multiresistant *K. pneumoniae* were susceptible to carbapenems [34].

The expression of major porins in *K. pneumoniae*, such as *ompK*35 and *ompK*36, can be altered due to genetic mutations resulting in either reduced porin expression or complete loss of porin expression [35]. The porins are outer membrane proteins that provide a channel for the entry of a wide range of antibiotics into the bacterial cell [35]. Porin loss was detected in six strains of bla_OXA-48_-positive CRKP, one strain of *bla*_NDM_-positive CRKP and two strains of non-carbapenemase-producing CRKP. It has been demonstrated that the level of carbapenem resistance was reduced significantly when functional *ompK*36 was restored in an AmpC-producing isolate that was *ompK*36-deficient in which a high level of carbapenem resistance was observed [35]. This indicated that carbapenem resistance in AmpC-producing-*K. pneumoniae* was associated with porin expression. In addition, it was also reported that isolates that harboured *bla*_OXA-48_ in combination with porin deficiencies showed high-level carbapenem resistance due to the association of *bla*_OXA-48_ with the reversely oriented insertion sequence, IS1999, which consequently resulted in the high production of OXA-48 [22]. 

In non-carbapenemase-producing CRKP, Gröbner et al. observed that the porin loss and overexpression of a multidrug efflux pump together with other unknown mechanisms contributed to carbapenem resistance [5]. His study revealed that the loss of *ompK*35 and/or *ompK*36 porin expression was due to various amino acid substitutions, point mutations resulting in a stop codon (TGA) and the transposition of insertion sequences into the coding region of porin genes, which resulted in a frameshift, and then generated a stop codon (TGA) [36]. Elsewhere, Yan et al. reported on non-carbapenemase-producing CRKP isolates that showed the overexpression of AmpC β-lactamases without any mutations in the *ompK*35 and *ompK*36 genes and suggested the possibility of the protein structure alteration, decreased expression or inactivation of porins *ompK*35 and *ompK*36 in these isolates [34]. 

PFGE is considered as the “gold standard” for bacteria typing [37]. Although whole genome sequencing (WGS) which provides more information has been proposed as an alternative method for bacteria typing, PFGE remains an affordable and relevant technique in small laboratories and hospitals, especially in developing countries. Due to its high discriminatory power, PFGE is useful in outbreak investigations, surveillance and infection control [38]. The comparison of the PFGE profiles of all CRKP strains in this study showed high clonal diversity, indicating that the acquisition of CRKP strains in patients was by chance, and no obvious clonal transmission was observed. No patient-to-patient transmission or acquisition from a common source could be observed. In contrast, Low et al. reported a high prevalence of clonal ST101 strains in the surgical ward UMMC from August 2013 to September 2013 based on the PFGE analysis [14]. This predominant pulsotype reported by Low et al. did not appear again in this study. Some factors that might have contributed to the local spread are the promiscuous use of antibiotics, delay in detection of index cases, poor infection control procedures and colonization of patients or healthcare workers who then become reservoirs for continued transmission [26,39]. 

The risk of CRKP acquisition is associated with antibiotics exposure [40]. Many reports have indicated that the emergence of carbapenemase-producing *Enterobacteriaceae* (CPE) was the result of antibiotic selection pressure induced by repeated exposures to antimicrobials, and that any class of antibiotics (not limited to carbapenems alone) can select for CPE, such as CRKP [7]. Ninety five percent of the patients in this study had been exposed to at least one antibiotic for 90 days prior to CRKP detection. Sixty three percent of the patients had been prescribed carbapenems in the past 90 days. Half of the patients with comorbidities had long-term exposure to various antibiotics, which very likely contributed to the gradual development of multidrug-resistant bacterial infection, including CRKP [41]. The average length of hospitalization among the patients was 53.2 days. Prolonged hospitalization and/or long-term antibiotic treatment facilitate colonization by *K. pneumoniae* in mucosal surfaces of the GIT and oropharynx, predisposing patients to infection by *K. pneumoniae* originating from their own microbiota [42,43]. The risk of CRKP infection is amplified in colonized patients who have undergone surgical procedures, not to mention patients with underlying comorbidities, such as cancer and diabetes mellitus [44]. 

In our study, central venous catheterization, CRKP colonization and NDM producers were significant confounding factors for in-hospital mortality with CRKP infections. Seventy five percent of the patients in this study had central venous catheter insertion. The use of central venous catheter was associated with a higher risk of in-hospital mortality. This finding was similar to the risk factors of CRKP infection reported from previous studies [45,46]. While colonization serves as significant reservoirs for CRKP transmission, contaminated fomites, such as catheters, scopes, implants, tracheal tubes and other medical devices, play an equally important role in the spread of *K. pneumoniae*, especially in ventilated and catheterized patients, since *K. pneumoniae* can attach itself firmly onto fomites by means of its fimbriae [47]. However, patients colonized with CRKP and NDM-producing CRKP had a lower risk of in-hospital mortality. 

Based on the multivariate Cox analysis, patients infected with NDM producers, unexpectedly, had a lower risk of death and longer survival times. NDM carbapenemase belongs to class B β-lactamases, also known as metallo β-lactamases. Its activity is inhibited by metal chelators [4]. Based on available clinical data, colistin is the most frequently used definitive treatment to treat CRKP infection in our hospital setting. Since most NDM producers retain their susceptibilities to colistin, fosfomycin and tigecycline [48], this might have contributed to the lower rate of in-hospital mortality observed in the patients infected with NDM producers. The association between in-hospital mortality and other carbapemase genes, such as *bla*_KPC_, *bla*_IMP_, and *bla*_VIM_, could not be further explored in this study due to their absence among the tested CRKP strains. 

Indeed, data on the molecular epidemiology of CRKP in Malaysia are still lacking, and their prevalence may very well be underestimated as much as it is for many countries in South East Asia, as previously noted in multiple reports [28,49,50]. As the current study was limited to CRKP strains obtained from our hospital within a short duration of time, the findings of this study can only represent the current assessment of CRKP existing in UMMC at the present time.

The emergence of CRKP as a significant aetiologic agent in healthcare-associated infections is an escalating public health threat, not only for Malaysia but all over the world. The successful spread of CRKP was contributed to greatly by the fact that *K. pneumoniae* itself is a notorious nosocomial pathogen capable of causing outbreaks in hospitals, in addition to other factors, such as inappropriate antibiotic use (not limited to carbapenems), overstretched healthcare systems, poor infection control, high-density populations, international travel and medical tourism [28]. Moreover, with the limited antimicrobials available to treat CRKP infections, reports on resistance even to those drugs as demonstrated by the detection of two colistin-resistant *K. pneumoniae* in this study highlight the critical need for the early identification and determination of genotypic characteristics of CRKP in order to enable the administration of accurate and appropriate antimicrobial therapy. It is of the utmost importance to undertake all measures necessary to combat the emergence of carbapenemase-producing bacteria in our setting, as these multidrug-resistant bacteria will undoubtedly and inevitably threaten future antimicrobial chemotherapy and contribute towards a more challenging path in the management of CRKP or other CRE infections in the future.

## 4. Materials and Methods

### 4.1. Ethics Statement

Clinical data of patients were retrieved from the hospital database with ethics approval from the University of Malaya Medical Centre Medical Ethics Committee in 2015 (MEC ID: 20154-1249).

### 4.2. Data Collection and Bacterial Strains 

This study was conducted in the University of Malaya Medical Centre (UMMC), a teaching and tertiary care hospital in Kuala Lumpur, Malaysia. A total of 63 non-duplicated clinical isolates of CRKP strains were collected within a twenty-month period. The inclusion criterion for carbapenem-resistant strains was defined as *K. pneumoniae* strains that were resistant or intermediate to at least one of the tested carbapenems (imipenem, meropenem and ertapenem) according to CLSI guidelines [51]. In a patient with multiple specimens in which CRKP was isolated, only the first occurrence was included in the study. Patients’ demographic, presence of indwelling devices, clinical, treatment and outcome data were retrieved from the electrical medical record (EMR). Patients were deemed to have an infection if there was the presence of clinical and biochemical evidence of an infection, and colonization if patients did not have any signs or symptoms and there was no evidence of infection [52]. The location of acquisition of CRKP was classified into hospital acquired (HA), community acquired (CA) and healthcare associated (HC). HA was defined as an isolation of CRKP from a patient >48 h after admission, who did not show any signs or symptoms during admission; CA referred to the isolation of CRKP from a patient <48 h after admission, who did not have contact with any healthcare system in the previous three months; HC was defined as the isolation of CRKP from a patient <48 h after admission, who had contact with a healthcare system in the previous three months, such as a nursing home, or those who were under regular haemodialysis, received recent intravenous antibiotic therapy or chemotherapy or were hospitalized in an acute care hospital for more than 2 days in the previous three months of isolation [53,54]. Previous antibiotic exposure was defined as antibiotics used in the last three months. Antibiotic treatment was divided into empiric and definitive antimicrobial therapy. Empiric antibiotic is defined as the initial antibiotic administered in the first 48 h after the onset of bacteraemia before the microbiological results, whereas definitive therapy refers to antibiotics given after the microbiological susceptibility results are available [55,56]. 

All bacterial strains were revived from stock culture and checked for purity before the commencement of laboratory work. Bacterial identification and antimicrobial susceptibility testing (AST) were performed by an automated system (Vitek^®^ 2; BioMérieux, Marcy L’Etoile, France). All the isolates were identified as *K. pneumoniae* by Vitek^®^ 2 using an ID-GN card (BioMérieux), and AST was performed using an AST-N314 card (BioMérieux). A Modified Hodge Test (MHT) was performed to detect carbapenemase production by using *K. pneumoniae* ATCC BAA-1705 and *K. pneumoniae* ATCC BAA-1706 as MHT positive and negative controls, respectively, in accordance with the Clinical Laboratory Standard Institute (CLSI) guidelines [51]. 

### 4.3. PCR Detection of Carbapenemase Genes and Porin-Associated Genes

Detection of carbapenemase genes, *bla*_KPC_ [57], *bla*_NDM_, *bla*_IMP_, *bla*_VIM_ [58] and *bla*_OXA-48_ [59] was performed via PCR amplification by using primers and cycling parameters as previously described [57,58,59]. Selected amplified products were sequenced and BLASTed against the NVBI nucleotide database using Standard Nucleotide BLAST (http://blast.ncbi.nlm.nih.gov/Blast.cgi accessed on 10 September 2017) to validate the identity. For the detection of porin-associated genes, *ompK*35, *ompK*36 and *ompK*37 [60], PCR amplification was similarly carried out using established primers and amplification conditions, as described by Kaczmarek et al. [60].

### 4.4. Pulsed-Field Gel Electrophoresis (PFGE)

PFGE was performed on CRKP strains to determine the clonal relatedness with the standard protocol from PulseNet [61]. Briefly, plugs containing genomic DNA were digested with XbaI restriction enzyme (Promega, Madison, WI, USA) and separated in a CHEF Mapper XA system. The PulseNet universal strain Salmonella enterica, serotype Braenderup H9812 was used as the reference standard marker. Interpretation was performed using Bionumeric software version 7.6.2. (Applied Maths, Sint-Martens-Latem, Belgium). A dendrogram was generated using dice correlation coefficients and clustered by the unweighted pair group method using arithmetic average (UPGMA) with a tolerance of 1.5%. More than 80% similarity upon dendrogram analysis was considered as one cluster.

### 4.5. Statistical Analyses

Patients’ data from medical records, including age, ethnic, gender, length of stay, hospital admission diagnosis, ward, site of infection, date of isolation, causative organisms, infection acquire model, invasive devices, prior hospitalization, co-morbidities, previous antibiotics exposure, administered treatments and treatment outcomes, were retrieved. Microbiological data, including strain susceptibilities to selected antibiotics, presence of carbapenemase genes and detection of porin loss genes, were recorded. 

Risk factors associated with in-hospital mortality were firstly compared using IBM SPSS ™ Statistic software, version 25. A chi-square test was performed for categorical variables, whereas continuous variables were analysed using a Mann–Whitney U test or Student’s t-test depending on the data normality. Parameters with *p* values less than 0.25 were selected for multivariate firth logistic regression to model the risk factors associated with in-hospital mortality. The model was first built using the brglm R package, and the parsimonious model was selected using the “dredge” command implemented in the MuMIn R package.

The Cox proportional hazards model was used to evaluate the relationship of the potential risk factors with the survival time. The univariate Cox regression was performed with the survival R package. The variable selection for multivariate mode was conducted using the My stepwise R package, in which the significance levels for entry (SLE) and significance level for stay (SLS) were set at 0.150.

## Figures and Tables

**Table 1:** Characteristics of carbapenem-resistant *Klebsiella pneumoniae* associated with in-hospital mortality and firth logistic results.

		In Hospital Mortality		*p* Value	OR (95% CI)
		Yes	No		
Gender	Female	8 (34.8%)	15 (65.2%)	0.292	
	Male	18 (48.6%)	19 (51.4%)		
Ethnic	Malay	6 (33.3%)	12 (66.7%)	0.246	
	Chinese	13 (50%)	13 (50%)		
	Indian	5 (35.7%)	9 (64.3%)		
	Others	2 (100%)	0		
Carbapenemase gene	No carbapenemase	3 (60.0%)	2 (40.0%)	0.118	
	OXA	17 (51.5%)	16 (48.5%)		
	NDM	3 (20.0%)	12 (80.0%)		0.081 (0.0067–0.43)
	OXA & NDM	3 (42.9%)	4 (57.1%)		
porin loss	No loss	22 (43.1%)	29 (56.9%)	0.163	
	Loss of *OMPK* 36	0	3 (100%)		
	Loss of *OMPK* 37	4 (66.7%)	2 (33.3%)		
Infection model	CA	2 (50.0%)	2 (50.0%)	0.003	
	HA	18 (60.0%)	12 (40.0%)		0.26 (0.17–1.66)
	HC	2 (100%)	0		
	Unsure	1 (100%)	0		
	Colonized	3 (13.0%)	20 (87.0%)		0.02 (0.00053–0.17)
ICU admission	No	11 (44.0%)	14 (56.0%)	0.93	
	Yes	15 (42.9%)	20 (57.1%)		
Co-morbidity	CKD	5 (62.5%)	3 (37.5%)	0.24	
	DM	9 (47.4%)	10 (52.6%)	0.668	
	Malignancy	1 (50.0%)	1 (50.0%)	0.847	
	Other diseases	14 (48.3%)	15 (51.7%)	0.455	
Invasive devices	Indwelling catheter	20 (48.8%)	21 (51.2%)	0.211	
	Mechanical ventilator	17 (44.7%)	21 (55.3%)	0.773	
	Tracheostomy	3 (37.5%)	5 (62.5%)	0.721	
	Central venous catheter	23 (51.1%)	22 (48.9%)	0.035	12.90 (2.23–210.37)
	Art line	13 (48.1%)	14 (51.9%)	0.496	
	Peripheral line	17 (40.5%)	25 (59.5%)	0.495	
	Nasogastric tubes	19 (45.2%)	23 (54.8%)	0.649	
	Stoma	5 (71.4%)	2 (28.6%)	0.11	
	Other	9 (50.0%)	9 (50.0%)	0.495	
Empiric treatment	Yes	17 (47.2%)	19 (52.8%)	0.457	
	No	9 (37.5%)	15 (62.5%)		
Anti CRKP treatment	Yes	15 (57.7%)	11 (42.3%)	0.05	
	No	11 (32.4%)	23 (67.6%)		
Monoinfection	Yes	6 (54.5%)	5 (45.5%)	0.406	
	No	20 (40.8%)	29 (59.2%)		
Total		26 (43.3%)	34 (56.7%)		

OXA: Oxacillinase, NDM: New Delhi metallo-β-lactamase, CA: Community-acquired, HA: Hospital-acquired, HC: Healthcare-aquired, CKD: Chronic kidney disease, DM: Diabetes mellitus, CRKP: carbapenem resistant *Klebsiella pneumoniae*.

**Table 2:** Cox proportional hazard model for mortality associated with CRKP infection.

Variables	Univariate Analysis			Multivariate Analysis		
	Hazard Ratio (HR)	95% CI for HR	*p* Value	Odd Ratio	95% CI for HR	*p* Value
Ethnic	0.78	0.53–1.2	0.22			
*Carbapenemase genes*						
OXA	2.5	1.1–5.6	0.029			
NDM	0.25	0.073–0.83	0.024	0.27	0.079–0.93	0.038
OXA & NDM	0.74	0.22–2.5	0.62			
No carbapenemases	1.6	0.46–5.3	0.47			
MHT	1.3	0.51–3.2	0.6			
*Porin-associated gene*						
No porin loss	1.1	0.39–3.3	0.82			
OMPK 36	3.8 × 10^−8^	0-Inf	1			
OMPK 37	1.2	0.39–3.4	0.79			
CRKP infection models						
HA	1.6	0.7–3.7	0.26			
CA	2.6	0.59–12	0.21			
Colonizer	0.23	0.069–0.76	0.016			
*Invasive devices*						
Indwelling catheter	1.8	0.72–4.6	0.2			
Central venous catheter	2.2	0.65–7.3	0.21			
Stoma	3.5	1.2–9.9	0.019	2.63	0.906–7.61	0.075
Co-morbidity of chronic kidney diseases	1.6	0.58–4.2	0.38			
*Previous exposure of antibiotics*						
Piperacillin-tazobactam	1.6	0.7–3.5	0.27			
Second-generation cephalosporin	0.43	0.1–1.8	0.25			
Third-generation cephalosporin	0.41	0.18–0.93	0.032			
*Empirical antibiotic treatment*						
Amoxicillin-clavulanate	1.9	0.57–6.6	0.29			
Meropenem/imipenem	1.8	0.79–4	0.17			
*Definitive antibiotic treatment*						
Meropenem	1.1	0.42–2.7	0.88			
Imipenem	2.2	0.9–5.4	0.084	2.21	0.898–5.44	0.084
Colistin	1.3	0.58–2.8	0.55			

Inf: Infinity.

## Data Availability

Not applicable.
